# Reviewing Strategies and Our Approach to Mapping and Ablation of Left Ventricular Summit Arrhythmias

**DOI:** 10.3390/jcm14176120

**Published:** 2025-08-29

**Authors:** Ziad Abuiznait, Mohamad Ghanayem, Nizar Andria, Ali Sakhnini, Edo Birati, Ibrahim Marai

**Affiliations:** 1 The Lydia and Carol Kittner, Lea and Benjamin Davidai Division of Cardiovascular Medicine and Surgery, Cardiovascular Department, Tzafon Medical Center, Poriya P.O. Box 15208, Israel; 2 The Azrieli Faculty of Medicine, Bar Ilan University, Safed P.O. Box 1589, Israel

**Keywords:** ventricular arrhythmia, left ventricular summit, ablation

## Abstract

**Background:** Left ventricular (LV) summit is an important origin for ventricular arrhythmias (VAs). However, the complex electroanatomic structure of LV summit and the surrounding anatomic sites makes ablation of this arrhythmia challenging. **Aim:** In this paper, we review the main strategies to mapping and ablation of LV summit VAs and summarize our experience in this challenging ablation. **Methods:** To summarize our experience, we included all consecutive patients with outflow VAs referred to our institute for ablation between 2019 and 2024 who were eventually diagnosed with LV summit origin based on electroanatomical mapping and ablation result using stepwise and sequential ablation approach. **Results:** A total of 38 patients were found to have VAs from LV summit origin. Overall five patients had history of at least one failed ablation. V1 transition was seen in 15 patients, V2 transition in 12 patients, and V3 transition in 11 patients. Four patients had R wave pattern break in lead V2. Ablation was performed from the earliest activation or from one of the adjacent sites using stepwise and sequential approach. Acute suppression of VAs occurred in 35 patients without complications, except one case of pseudoaneurysm of femoral artery. **Conclusions:** Stepwise and sequential ablation approach can suppress VAs originating from LV summit in most patients.

## 1. Introduction

Idiopathic ventricular arrhythmias (VAs), including ventricular premature contractions (VPCs) and ventricular tachycardia, are common arrhythmias. These arrhythmias can be benign, without any symptoms or consequences, but they can also cause severe symptoms and lead to arrhythmia-induced cardiomyopathy, mainly when the burden of the arrhythmia is high. Antiarrhythmic drugs are not always effective and may have proarrhythmic effect. However, catheter ablation of idiopathic VAs is highly successful and is accepted as a first-line therapy or as an alternative for antiarrhythmic medications, according to current guidelines [1]. Overall, the success rate is about 90%. However, anatomic and or electrophysiologic limitations can reduce the success rate in some patients. VAs originating from papillary muscles, para-Hisian area, and left ventricular (LV) summit are examples of challenging sites for mapping and ablation and relatively low success sites compared to highly success sites, such as right ventricular outflow tract (RVOT), despite the advances in mapping and ablation techniques in the last years [1]. LV summit is the highest portion of the LV epicardium, located near the bifurcation of the left main coronary artery (LMCA), and accounts for up to 14.5% of LV VAs [2]. LV summit is an important origin for VAs, including VPC and ventricular tachycardia. However, the complex electroanatomic structure of LV summit and the surrounding anatomic sites makes ablation of this arrhythmia challenging. The likely reason for the limited success of catheter ablation for these VAs is that their site of origin is subepicardial and/or intramural. Understanding the anatomy of LV summit and the adjacent anatomic structures, as well as using different mapping techniques, are crucial for localization of the origin of the arrhythmia and the target site for ablation [3,4].

In this paper, we review the main strategies for the mapping and ablation of LV summit VAs and summarize our experience in ablation of these challenging arrhythmias.

## 2. Anatomy of LV Summit

LV summit is a triangular region situated in the most superior part of the left epicardial ventricular region and covered more or less by epicardial isolating layers of fat [5]. This triangular region is bounded by the bifurcation between the left anterior descending (LAD) and the left circumflex (LCx) coronary arteries and is bisected laterally by the great cardiac vein (GCV), resulting in two regions: an area inaccessible (medial and more superior region, close to the apex of the triangle) to catheter ablation because of close proximity to the major coronary vessels and the presence of epicardial fat; and an area accessible to catheter ablation, which is a more lateral and inferior region, located toward the base of the triangle [2]. The endocardial site, which is against to LV summit, is the basal portion of the LV ostium next to the septum, and it is in direct contact with the left coronary cusp (LCC). The LCC forms the lateral and posterolateral attachment of the aorta to the LV ostium, and it is in close proximity to the intramural component of the LV summit [6]. On the other side, the anteroseptal aspect of the right ventricular outflow tract (RVOT) is adjacent to the LCC, in close proximity to the LAD and anterior interventricular vein (AIV), whereas its posteroseptal aspect is adjacent to the right coronary cusp [6].

The myocardium bounded between the epicardium of LV summit and LV endocardium below aortic cusps can be a source of arrhythmias, referred to as LV summit VAs. Based on the anatomical definition of the LV summit, LV summit VAs can have different electrophysiologic characteristics and different anatomical approaches for ablation. These arrhythmias can be targeted directly by ablating the exact origin or indirectly by targeting the adjacent structures and anatomic vantage points, including left ventricular outflow tract (LVOT), LCC, GCV/AIV, RVOT, or pulmonic cusps [7]. The septal branches of the LAD and the septal veins draining into the AIV could also be used for mapping and ablation of these arrhythmias [6]. Importantly, anatomical variations of the LV summit area and adjacent structures are not rare. Anatomic variations of the coronary venous systems could impact the ability to advance mapping and ablation catheters to GCV-AIV junction and septal branches as well as the ability to deliver radiofrequency (RF) energy based on the distance of coronary veins from coronary arteries. Variations in coronary vessels could also cause different effects of RF due to the heat sink effect, which can reduce the effectiveness of ablation due to the cooling effect of blood flow. In addition, the thickness of epicardial fat and the thickness of the myocardium can impact the ability to achieve transmural lesion.

## 3. ECG Characteristics of LV Summit

Given the close proximity of LV summit to various anatomical structures, there is significant overlap of electrocardiographic pattern of VAs originated from LV summit, LVOT, aortomitral continuity, aortic cusps, coronary venous system, and RVOT [8,9,10,11]. LV summit VAs typically have a right bundle branch block (RBBB) pattern with inferior axis and larger R waves in lead III compared to lead II, but a left bundle branch block (LBBB) pattern with inferior axis and early transition (V2 or V3) also can be seen [2]. A QS pattern in lead I is observed in 30% of patients [6]. Another unique ECG pattern is the precordial “pattern break” in V2, consisting of an abrupt loss of R wave in lead V2 compared to V1 and V3 [11]. This suggests an origin from the septal aspect of the LV summit (anatomically opposite to lead V2), usually in close proximity to the LAD and less likely to be eliminated from the epicardial aspect. Proximity to LAD precludes ablation in about half of patients. Long-term VA suppression could be achieved in only 58% of cases most commonly when the earliest site is at the anterior and leftward RVOT just under the pulmonic valve (PV) [11]. Left bundle branch morphology and an abrupt R-wave transition in lead V_3_ indicating the septal margin of LV summit was also reported. In total, 80% of the outflow VA with abrupt R-wave transition in lead V_3_, which overwhelmingly exhibited the earliest activation from the epicardium or mid-myocardium, was eliminated by ablation from the vantage point of the interleaflet triangle below the right-left coronary junction, as reported by Liao et al. [12].

## 4. Mapping

**Figure 1:**
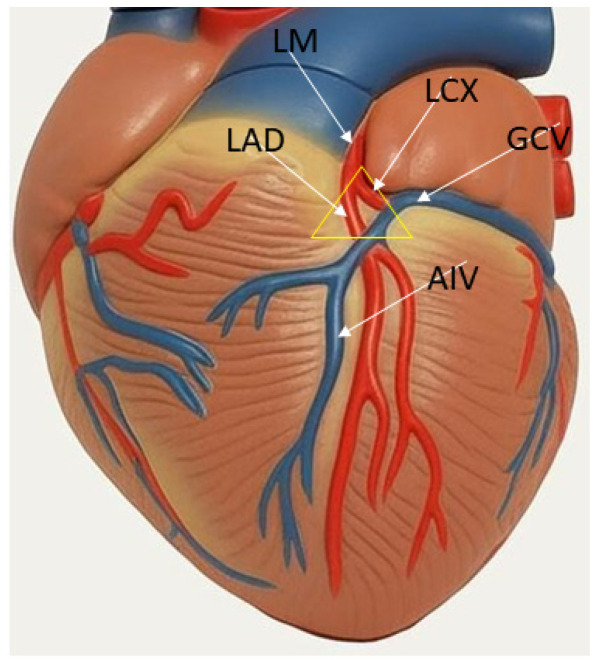
Anatomy of LV summit. LV summit (yellow triangle) is a triangular region situated in the most superior part of the left epicardial ventricular region. The triangle is bounded by the bifurcation between the left anterior descending (LAD) and the left circumflex (LCx) coronary arteries and is bisected laterally by the great cardiac vein (GCV). Adjacent structures to LV summit include aortic cusps, LVOT, RVOT, pulmonic cusps, and GCV-AIV (anterior interventricular vein) junction. These structures can be used to target LV summit VAs. LM—left main.

Because of the complex anatomy of LV summit, the highly variable anatomic relationships, and the overlapping of ECG patterns with adjacent structures, it is important to perform detailed electroanatomical mapping of LV summit and all structures that are in close proximity or have an anatomical connection to LV summit (Figure 1). RVOT and pulmonic cusps should be mapped. This is important not only for looking for early activation, but also for potential anatomic sites for ablation if LV summit ablation is unsuccessful or, if it is not possible to deliver energy in the left endocardial side or within coronary venous system because of the close proximity of major coronary arteries or in case of intramural origin of outflow arrhythmia. The coronary venous system should also be mapped using an advancing mapping catheter deep into coronary sinus (CS) to GCV- AIV junction. In many cases, the ablation catheter cannot be advanced deep in the coronary venous system if the diameter of the vessels is small. Diagnostic catheters with small diameters can be used for this purpose, and they can also be used for anatomical reference during anatomical ablation from RVOT or LVOT facing the earliest activation at the GCV-AIV site. Recently, wires have been used for unipolar mapping. Wires can be advanced deep into AIV septal branches. These wires can also serve as reference for anatomical ablation or can be used for ablation.

Then, the aortic cusps by retrograde approach as well as the LVOT by retrograde or trans septal approach, should be mapped in detail. The LVOT should be carefully mapped, including the area below the coronary cusps, aortomitral continuity, and lateral LVOT area. The area just below the aortic cusps is a challenging area for mapping and can be missed. A combined retrograde transaortic approach and antegrade transseptal approach with a reversed S curve of the ablation catheter may be needed to reach the anterosuperior LVOT below the aortic cusps, as reported by Ouyang et al. [13]. Intracardiac echocardiography (ICE) can help understand and define the real-time anatomy of LV summit and mark the important sites of the LV summit and adjacent sites during mapping and ablation. Depending on the 3-dimensional (3D) mapping system used, a 3D anatomic map of the ventricles, outflow tracts, aortic cusps, and pulmonic cusps can be constructed using ICE and dedicated modules. This 3D anatomic map can be integrated into and displayed on the 3D electroanatomical map [14]. Cardiac computed tomography (CT) can also be used and integrated into 3D mapping systems to better understand the complex anatomy of RVOT, LVOT, coronary arteries, and coronary venous system [15,16]. However, its impact on the safety and effectiveness of ablation is not known.

The aim of mapping is to define target sites for ablation. It is important to realize that early activation during VPCs and or perfect pace-mapping do not always exist, and even if they do exist, ablation is not always possible or effective due to proximity to coronary arteries, presence of epicardial fat, or significantly high impedance, as in the coronary venous system or small coronary venous branches that prevent advancing of the ablation catheter to the target site. In these situations, the ablation can be performed indirectly by targeting adjacent sites; thus, it is very important to precisely define the surrounding structures of the target site.

## 5. Ablation

As the LV summit is the highest point of the LV epicardium, it can be accessed directly via percutaneous pericardial puncture, but this approach is usually limited by the proximity to major coronary arteries and the presence of a thick layer of epicardial fat; however, it should be considered if other strategies failed and the ECG pattern and activation mapping suggest an origin from the accessible epicardial area. Santegali et al. [9] reported a total of 23 consecutive patients with VAs arising from the LV summit who underwent percutaneous epicardial instrumentation for mapping and ablation because of unsuccessful ablation from the coronary venous system and multiple endocardial LV/right ventricular sites. Successful epicardial ablation was achieved in only five (22%) patients. A Q-wave ratio of >1.85 in aVL/aVR, a R/S ratio of >2 in V1, and absence of q waves in lead V1, was associated with successful epicardial ablation [9]. Possible complications of epicardial access and ablation include inadvertent right ventricular puncture, pericardial effusion and tamponade, acute damage to coronary arteries and veins, and late coronary artery stenosis.

Alternatively, LV summit can be mapped and ablated using the stepwise and sequential approach. This approach is the cornerstone of LV summit ablation [6,7]. It is based on the anatomical approach if the earliest site cannot be directly ablated due to close proximity to coronary arteries, or if there is no early activation found after detailed mapping, as when all sites are found equally early, intramural origin is suggested.

LV summit VAs can be targeted from the coronary venous system (GCV/AIV, septal veins) if early activation is found. Ablation should be attempted within the venous system if the earliest activation is recorded at the GCV/AIV, and energy can be safely delivered [6]. Safe distance (at least 5 mm) from coronary artery should be confirmed by coronary angiography. Endocardial ablation may be performed sequentially from adjacent structures, such as the LCC, LVOT endocardium just below the aortic cusps, aortomitral continuity, anteroseptal RVOT, or pulmonic cusps; the option which is earliest and/or opposite to the earliest epicardial site should be used in the case of failure due to deep intramural site, inability to deliver energy within the coronary venous system due to proximity to coronary arteries, high impedance, or small vein diameter, or if the earliest activation is recorded at a septal venous perforator. Importantly, safe distance from LCC should be confirmed as needed based on coronary angiogram, integrated CT scan, or ICE based on institutional workflow.

For success of this “anatomical” approach, it is not mandatory to have optimal local activation time or good pace-mapping. An anatomic distance (<13 mm) between the earliest ventricular activation site in the coronary venous system and endocardial ablation site could be a predictor of a successful endocardial catheter ablation of LV summit VAs [17]. Frankel et al. [18] described for the first time successful elimination of ventricular arrhythmias arising from the LV summit AIV region with ablation from the nearby RVOT. The anatomic separation between the earliest RVOT and AIV sites was less than 10 mm. The most anterior and leftward RVOT just under the PV was the site of successful ablation in patients with precordial “pattern break” in V2, suggesting an origin from the septal aspect of the LV summit [11]. In some cases, ablation above the pulmonic valve may be needed, as early activation could be present above the valve level given the anatomic proximity between the pulmonary artery and LV summit. Ablation at this site should be performed carefully, using the reversed U technique to avoid damage to left coronary system.

In general, an endocardial anatomic approach for LV summit VAs is most commonly successful in the LVOT, followed by the aortic cusps, and is rarely successful in the RVOT [17]. Inaccessible LV summit area (the most superior aspect of the LV summit region) can be anatomically targeted from subaortic region below LCC and or RCC-LCC junction and from left pulmonic cusp. Anecdotally, ablation of an LV summit ventricular tachycardia from the left atrium has also been reported [19]. Jauregui Abularach et al. [8] reported that the “anatomic” approach would be successful in more than half of cases. The stepwise anatomical approach was found to be feasible and effective in larger studies. Recently, Enriquez et al. [20] reported a stepwise anatomical approach to ablation of intramural outflow tract VAs guided by septal coronary venous mapping. The intramural origin was confirmed by demonstration of earliest activation in a septal coronary vein. RF ablation was performed from the closest endocardial site in the LVOT and/or RVOT, independent of the local activation time. If there was no suppression by endocardial ablation, retrograde transvenous ethanol infusion with a single or double balloon technique was performed, targeting the earliest septal coronary vein. If venous anatomy was not suitable for ethanol ablation or if this failed, bipolar ablation was performed. In 87% of cases (52 patients), endocardial ablation from the endocardial LVOT, RVOT or both was successful in eliminating the PVCs. In the remaining eight patients, the PVCs were eliminated with ethanol infusion (7 patients) and bipolar ablation (1 patient). Multicenter series included 92 patients with intramural outflow tract PVCs defined by earliest ventricular activation recorded in a septal coronary vein or ≥2 of the following criteria: (1) earliest endocardial or epicardial activation <20 ms pre-QRS; (2) Similar activation in different chambers; (3) no/transient PVCs suppression with ablation at earliest endocardial/epicardial site [21]. In 32% of patients, direct mapping of the intramural septum was performed using an insulated wire or multielectrode catheter. Most patients required special ablation techniques (one or more), including sequential unipolar ablation in 73%, low-ionic irrigation in 26%, bipolar ablation in 15%, and ethanol ablation in 1%. Acute PVCs suppression was achieved in 75% of patients.

RF ablation with stepwise increments of RF energy (target 20–40 W) is usually performed using of irrigated catheters. Irrigation is mandatory to deliver adequate power in the low-flow venous system. Low-ionic irrigation, which was required in 26% of 92 patients in a multicenter study with intramural outflow tract VAs, can help to achieve transmural lesions [21].

## 6. Special Ablation Techniques

Special techniques maybe needed in many cases after failure of standard RF ablation. Special techniques can be used as part of stepwise/sequential approaches, or as a main, primary technique.

### 6.1. Prolonged Duration Ablation

The proximity of major coronary arteries and presence of fat generally precludes direct epicardial ablation. Transient suppression of VAs is a well-known issue after ablation. VAs may recur within a few minutes after ablation, indicating only non- transmural lesion. An alternative strategy for targeting epicardial or intramural LVs VA is to create large lesions from adjacent locations, particularly the endocardium of the subvalvular LVOT and/or the aortic cusp region, by delivering incremental RF energy over an extended duration (>120 s) [22]. Garg et al. [22] reported their experience with prolonged duration endocardial ablation strategy compared to standard ablation. For VAs suspected and/or confirmed to originate from the LV summit region, energy was delivered from the lowest aspects of aortic cusp region and/or the subvalvular LVOT (≤0.5 cm from the valve). Lesions were typically created in clusters over the area demonstrating earliest activation and/or surrounding the site with the best pace-map matches beyond 120 s up to a maximum of 5 min, if standard RF ablation (60–120 s) was unable to consistently suppress the VAs. Power delivery was attenuated and/or terminated if rapid impedance dropped, any impedance rose, and/or rapidly expanding echogenicity (seen with ICE) during lesion creation was noticed. Short-term clinical success was achieved in 60% of patients with standard and in 79% of patients with prolonged duration RF ablation (*p* = 0.04) without differences in complications.

### 6.2. Bipolar Ablation

Bipolar ablation can overcome some of the problems of ablation of LV summit arrhythmias and increase the chance of achieving a transmural lesion. Bipolar RF ablation involves the application of alternating current between two ablation catheters positioned on opposite sites of the arrhythmogenic substrate, with one connected to the active port of the RF generator (active catheter), and the second connected to the indifferent port instead of the skin dispersive “ground” patch (return catheter) [23,24]. Consequently, RF current flows between the distal tip of both catheters, resulting in higher current density to the myocardial tissue and increased lesion transmurality [25]. The presence of multiple sites surrounding LV summit, which are accessible to classic ablation approaches, provides a broad spectrum of configurations for bipolar RF ablation, leaving the field clear for numerous approaches.

For anteromedial LV summit area, bipolar ablation can be performed between two ablation catheters: the first ablation catheter is positioned in the RVOT and serves as a return electrode during bipolar RF ablation, and the second, open irrigated ablation catheter is located in the subaortic region of LVOT. Such an approach was performed successfully in four patients without complications, and with a notable success rate of 75% [26]. For ablation of inaccessible LV summit, bipolar RF can be anatomically performed between two open-irrigated ablation catheters; the first ablation catheter (acting as return electrode) is positioned in the left pulmonic cusp, and the second ablation catheter is located in the subaortic region of LVOT. Positioning ablation catheter into left pulmonic cusp can be performed using the reversed U-shape technique, which allows positioning of the tip of the ablation catheter anatomically below the level of the proximal LAD artery [27,28]. This bipolar RF anatomic approach resulted in VA suppression in five of seven patients with inaccessible LV summit VAs refractory to conventional RF catheter ablation without complications [29]. For bipolar RF ablation of more lateral LV summit aspect (earliest local activation in the GCV or AIV), an active catheter is located in LVOT or RVOT at the site of earliest endocardial activation or the site anatomically closest to the earliest epicardial site, whereas a return catheter is positioned in the GCV/AIV [23,30,31]. This bipolar RF ablation approach led to acute VAs elimination in all patients after failed unipolar ablation from sites of early activation (AIV/GCV, LVOT, aortic cusps, and RVOT) without procedural-related complications [23]. In general, bipolar ablation was required in 15% of 92 patients in a multicenter study with intramural outflow tract VAs [21].

### 6.3. Ethanol Infusion

Retrograde coronary venous ethanol Infusion is recently used for ablation of refractory LV summit VAs [32,33]. This approach requires detailed understanding of the individual coronary venous system anatomy. This approach was pioneered by Dr Valderrábano and his group [32,34,35]. Briefly, as described by Dr Valderrábano, when the earliest signals are detected in the region of the GCV/AIV junction after initial mapping, if necessary, vein targets can be defined by accessing the coronary sinus via the femoral vein with a sheath, through which a multielectrode catheter is used to map the GCV and AIV. The earliest sites in the GCV or AIV are defined. The multielectrode catheter is then removed, and an angioplasty guide catheter is advanced to perform venograms to identify suitable intramural vein branches near the earliest site. Once identified, an angioplasty wire is advanced selectively in the targeted branch and configured as a unipolar catheter by covering the wire with an angioplasty balloon, leaving approximately 5 mm of the wire tip exposed. Local activation time in AIV branches and pace map correlations are used to define vein branches to be targeted based on earliest timing and best pace maps. Once a vein branch is defined as the target vein, the angioplasty wire is removed. Contrast is injected via the angioplasty balloon to assess the size of the branch and the extent of the targeted tissue reached (myocardial staining). Up to seven injections of 1 mL ethanol followed, depending on the therapeutic response and the extent of myocardial staining. The same group have also developed a double-balloon technique for large substrate ablation [36].

Retrograde coronary venous ethanol infusion offered a significant long-term effective treatment for patients (76% of patients had LV summit origin) with drug and RF-refractory VAs based on a multicenter experience [35]. Enriquez et al. [20] reported that ethanol infusion was needed to eliminated intramural outflow VAs in 7 out of 60 patients. Hanson et al. [21] reported that ethanol infusion was required in 1% of 92 patients in a multicenter study with intramural outflow tract VAs.

### 6.4. Wire Ablation

An epicardial LV summit origin is suggested when the ventricular activation time at the distal GCV or proximal AIV is earlier than other sites within the LVOT or RVOT. Mapping of the septal perforator vein using guidewire allows VAs originating in the epicardium (earlier in GCV/AIV) to be distinguished from those originating in the intramural foci (earlier in septal perforator), so the next mapping efforts can be directed accordingly. Efremidis et al. [37] reported two cases of RF ablation through a guidewire in the distal GCV/AIV using low energy protocols (10–20 W, impedance drop of 10 ohms for 20–30 s). RF was delivered by placing the proximal end of the guidewire in a saline bath along with the ablation catheter [37]. Another case was reported in which an angioplasty guidewire was used for successful mapping and ablation of LV summit VPCs within the distal part of GCV [38]. In this case report, RF was delivered by leaving approximately 25 mm of wire tip exposed for ablation and connecting the end part of the wire to the RF ablation.

Finally, surgical ablation has been described as an option for patients who do not respond to endocardial and epicardial ablation [39,40].

## 7. Our Approach

All patients with outflow VAs during 2019–2024 were retrospectively reviewed. Patients found to have VAs originating from LV summit were included in this study. The diagnosis of LV summit VAs was based on ECG pattern and results of ablation. VAs with ECG patterns of inferior axis and RBBB pattern or LBBB and early precordial transition (at or less V3) or break pattern at V2 were suspected to originate from LV summit. LV summit was confirmed during procedure if the earliest activation was at distal GCV or GCV-AIV junction (epicardial LV summit) or septal veins (intra mural LV summit), or if no early site was found after detailed mapping, or if all adjacent sites were found equally early, suggesting intramural origin.

### 7.1. Procedure

In general, electroanatomic mapping of the entire outflow tracts during point-by- point catheter manipulation was performed using the CARTO (Biosense Webster) system. First, mapping of RVOT and pulmonic cusps was performed, and then the coronary venous system, including GCV-AIV and or septal veins, was mapped. The mapping of the aortic cusps and LVOT is performed by retrograde approach. If LV summit origin was suspected based on the mentioned criteria, or if standard endocardial ablation failed to suppress the VAs, then stepwise/sequential ablation approach was performed until VAs were suppressed. Ablation would be performed first at GCV-AIV if early activation was confirmed at this epicardial site. If ablation within the coronary venous system was not feasible or not successful, then ablation proceeded in adjacent sites, mainly LVOT and, if needed, LCC, based on early activation and on anatomic distance from the earliest epicardial site. When intramural LV summit/septal margin of LV summit was suspected based on ECG pattern and or mapping, then ablation would be attempted at LVOT at the earliest site just below aortic cusps (LCC or the interleaflet triangle below the right-left coronary junction) and, if needed, above the valve. If left side ablation failed or transiently suppressed the VAs, then ablation from RVOT (mainly at the most anterior and leftward part just below pulmonic valve) or the left pulmonic cusp was performed. Importantly, prolonged duration endocardial ablations were used if needed, as previously described [22]. Percutaneous epicardial ablation was used only when epicardial origin was suspected and after failure of ablation from coronary venous system and endocardial ablation from LVOT/RVOT.

Our standard approach for RF ablation using irrigated catheters involves 20–25 W for 30–60 s in coronary veins, 40–45 W for 120–240 s in LVOT, 25–30 W for 60 s in aortic cusps, 30–35 W for 90 s in RVOT, and 25–30 W for 60 s in pulmonic cusps. The target of ablation was to suppress VA within the initial 30 s, and to achieve and, if possible, maintain 10% impedance drop from baseline.

Acute procedural success was defined as elimination of VAs for at least 30 min. Clinical success was defined as >80% reduction of VA burden during the 12 months follow-up.

The study was approved by the local institutional ethics committee and conducted according to the Declaration of Helsinki. Informed consent was waived because of the retrospective nature of the study.

### 7.2. Statistical Analysis

Continuous variables were presented as mean and standard deviation or as range and median. Categorical variables were presented as numbers and percentages.

## 8. Results

### 8.1. Patients’ Characteristics

All consecutive patients with outflow VAs referred to our institute for ablation between 2019 and 2024 who were eventually diagnosed with LV summit origin were included in this cohort. Of 178 patients who underwent catheter ablation for outflow VAs, 38 (21.3%) patients were found to have VAs from LV summit origin: 37 patients had VPCs and 1 had ventricular tachycardia. Five patients were treated after at least one failed ablation. Baseline characteristics are summarized in Table 1. Transition was seen in V1 in 15 patients, V2 in 12 patients, and V3 in 11 patients. Four patients had R wave pattern break in lead V2.

### 8.2. Ablation Sites

**Figure 2:**
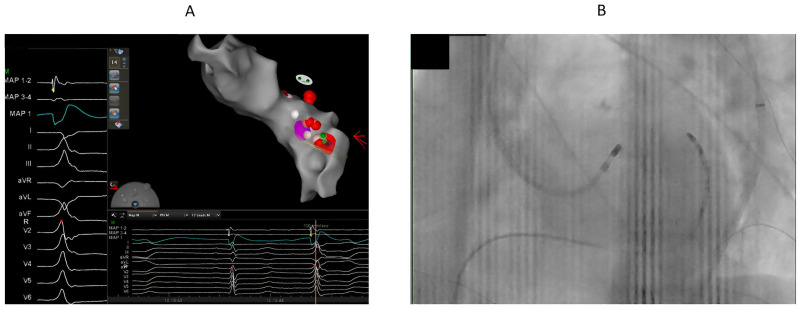
68-year-old male with high burden VPCs (RBBB pattern and inferior axis) and mildly reduced left ventricular function. (**A**). The earliest activation (19 ms before QRS) was at LVOT below and lateral to left coronary cusp. We could not advance catheter to GCV-AIV. We supposed that the origin might be close to GCV-AIV. Cluster ablation (red dots) for prolonged duration (240 ms) at lateral part of LVOT summit was performed, which eventually eliminated the VPCs. (**B**). The catheter ablation at the successful ablation site at LVOT.

**Figure 3:**
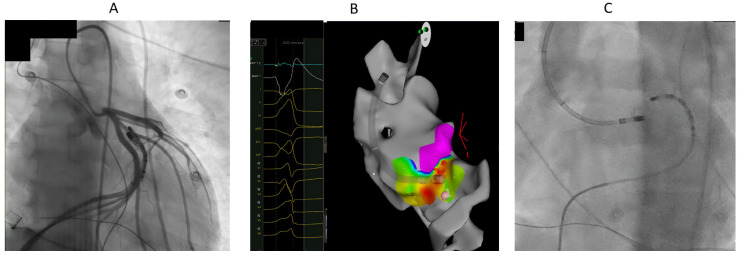
33-year-old male with high burden VPCs with V3 transition and inferior axis. (**A**). The earliest activation was at GCV-AIV junction. However, ablation was judged to be unsafe in this site because of proximity to coronary arteries. Ablation at LVOT adjacent to earliest site failed to eliminate VPCs in first ablation. (**B**). In redo procedure, prolonged duration (240 ms) anatomical ablation at LVOT adjacent to earliest site at GCV-AIV, as shown by electroanatomical mapping, successfully eliminated the VPCs. (**C**). Fluoroscopic image showing the ablation catheter in LVOT adjacent to diagnostic catheter at GCV-AIV.

Table 2 summarizes the sites of ablation. In total, 17 patients had successful ablation from LVOT below LCC or the right-left coronary junction (Figure 2 and Figure 3). Sequential ablation at LCC and LVOT was successful in eight out of nine patients. Sequential ablation from LVOT/LCC and RVOT/LPC was successful in seven patients.

The local endocardial electrogram at the sites of endocardial ablation preceded QRS less than 20 ms in all patients. Ablation was performed at these endocardial sites, as the early activation was found in these sites, or ablation at the early activation site within the coronary venous system was not found to be feasible. All patients needed prolonged endocardial ablation duration (more than 120 ms) for sustained suppression of VAs.

**Figure 4:**
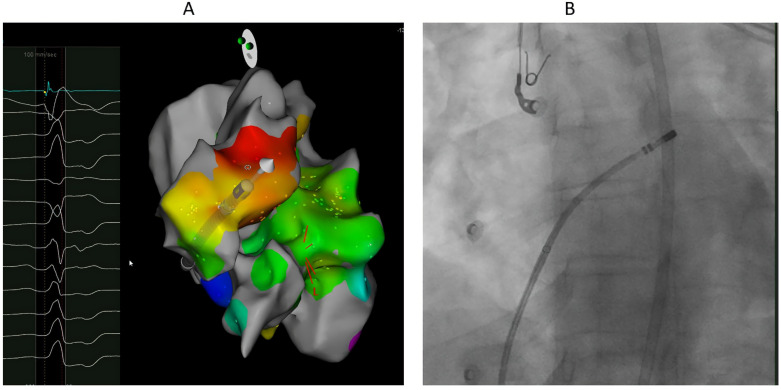
40-year-old female with high burden VPCs with R wave pattern break in V2. (**A**). Local electrogram at ablation catheter located at the anterior and leftward RVOT just under the pulmonary valve preceding QRS by 19 ms, which was earlier than sites from LVOT and GCV-AIV. Ablation at this site eliminated the VPCs. (**B**). LAO fluoroscopic projection showing the ablation catheter at the most anterior and leftward RVOT, just under the pulmonary valve.

Ablation from GCV-AIV after failure of ablation from LVOT eliminated VA in one of the two patients in which it was attempted. The baseline impedance within the coronary venous system was about 160–180 ohms without significant impedance issues during ablation. Ablation from RVOT only (at the anterior and leftward RVOT just under the pulmonary valve) was successful in one patient with R wave pattern break in V2 (Figure 4). Percutaneous epicardial ablation was attempted in two patients after failed endocardial ablation, but it failed to eliminate the VA in one of them.

Mean procedure time was 107 ± 39.4 min, fluoroscopy time was 13.8 ± 8.1 min, and total RF time was 420–1200 s (median 840 s). Complications were not reported, except for pseudoaneurysm of the femoral artery treated by local thrombin injection.

### 8.3. Clinical Follow Up

All patients who had acute suppression of VA sustained >80% reduction of VA burden during the 12 months follow-up, except one. The patient who had failed percutaneous epicardial ablation was treated with flecainide with control of VA. One patient who had failure of endocardial ablation at LCC/LVOT had significant reduction (>80%) of VA burden after 1 year of follow-up.

## 9. Discussion

Stepwise mapping approach and sequential ablation approach are effective to suppress VAs originating from LV summit area. Complications were not reported, except vascular complication in one patient.

Ablation of LV summit VAs is challenging. There are many approaches and strategies reported in the literature, as mentioned earlier. In general, all these strategies are based on deep understanding of the anatomy of LV summit and surrounding sites. ECG patterns can give us clues to the site of successful ablation: GCV/AIV when RBBB pattern exists; the vantage point of the interleaflet triangle below the right-left coronary junction when the pattern of left bundle branch morphology and an abrupt R-wave transition in lead V3 exists; and the most anterior and leftward RVOT below the left pulmonic valve when R wave pattern break in lead V2 exists. However, it is crucial to perform detailed electroanatomical mapping of all sites that are related and adjacent to LV summit as there is overlap of ECG patterns, and it is important for the sequential approach to be applied. The stepwise and sequential approach is key to successful ablation regardless of the ablation technique used. Special techniques may be needed in a significant number of patients, like prolonged duration, as in our cohort, or bipolar ablation or alcohol venous ablation, as reported in other studies.

**Figure 5:**
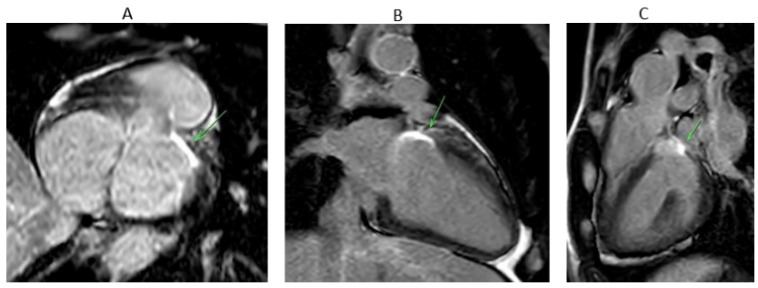
MRI images in orthogonal planes from a patient (the patient from Figure 2) who underwent endocardial LVOT prolonged duration ablation for left ventricular (LV) summit ventricular arrhythmia, demonstrating focal transmural late gadolinium enhancement (LGE) at the LV summit—corresponding to the most basal segment of the anterior LV wall. The green arrow highlights the area of LGE. (**A**) Short-axis view. (**B**) Two-chamber view. (**C**) Modified three-chamber view traversing the LV summit.

The aim of these techniques is to achieve a transmural lesion that can be detected by cardiac magnetic resonance imaging (MRI) (Figure 5). Preclinical and early clinical studies have shown that late Gadolinium enhancement (LGE)-MRI identified lesion volume and depth correlate with histopathology and, in some cases, with electroanatomic mapping during repeat procedures [41,42]. For LV summit ablation, where catheter stability, contact force, and transmurality are often suboptimal due to anatomical constraints, such imaging may offer valuable insights into long-term lesion durability and efficacy. Although promising, LGE-MRI lesion assessment in the ventricle has yet to be validated as a routine clinical endpoint. For electrophysiologists managing LV summit VA, integrating follow-up LGE-MRI may improve procedural evaluation, guide re-intervention, and refine long-term risk stratification.

Many patients may need reablation. Bipolar ablation and alcohol infusion within venous system are promising techniques that may be needed if other “standard approaches” fail to suppress VAs. In real world multicenter registry, bipolar ablation appeared safe and was feasible and effective in the majority of patients with refractory VA [24]. In this study, more than 70% of recurrent arrhythmias originated in the LV summit area, which highlights both the high prevalence of recurrent arrhythmias in the LV summit and the importance of some more advanced ablation techniques in that region. However, these techniques need special tools and appropriate training that is not yet available in most centers. In addition, randomized studies are needed to determine if these techniques are more effective than other techniques, like prolonged duration ablation. Every center should build its own mapping strategy and ablation technique based on the availability of mapping systems, imaging tools, and the expertise of operators. In addition, operators should seek to be familiar with special techniques, such as bipolar ablation and alcohol infusion. In our cohort, bipolar ablation and alcohol infusion techniques were not used. Thus, the results of this study could be relevant to many centers that do not use these special techniques.

Other energy sources, like pulsed field ablation (PFA), which recently emerged as a new treatment option for atrial fibrillation [43,44,45,46], may be used in the future in ablation of LV summit arrythmias. PFA is a non-thermal ablation modality highly specific for cardiac muscle, resulting in less collateral damage in other tissues, including coronary arteries [47,48]. Accordingly, PFA may result in transmural lesions, which will be effective in intramural foci without causing damage to coronary arteries. Benali et al. [49] reported two cases of PVCs originating from the LV summit region, responsible for severe PVC-induced cardiomyopathy, successfully treated with PFA using the lattice-tip catheter after multiple failed RF ablation procedures and ethanol infusion. Spenkelink et al. [50] reported a case of a patient with symptomatic epicardial LV summit PVCs underwent successful PFA via a subxiphoid approach, where RF ablation could not be used due to the proximity of the coronary arteries.

Our study has limitations. It is a single center retrospective study, which limits the ability to establish causation or control for confounding factors and reduces generalizability to broader or more diverse populations. In addition, the small sample size limits statistical power and external validity. Finally, localization of successful ablation site and clinical outcome prediction based on ECG patterns were not analyzed due to the small size of the study.

In summary, stepwise mapping and sequential ablation approach with prolonged ablation duration can suppress VAs originated from LV summit in most patients. Randomized studies are needed to compare this approach to other techniques.

## Figures and Tables

**Table 1:** Patients’ characteristics.

	N = 38
Age: years	57 ± 12.3
Males (%)	25 (65.8)
Hypertension (%)	19 (50)
Diabetes mellitus (%)	15 (39.5)
Coronary artery disease (%)	10 (26.3)
Ejection fraction (%)	50.34 ± 11.63
Burden of ventricular premature contractions (%)	21.4 ± 3

**Table 2:** Sites of ablation.

	Total = 38	V Transition by ECG	Failure = 3	Comments
LVOT below LCC	17	V1 = 9	0	
		V2 = 3	0	
		V3 = 5	0	
LCC+ LVOT	9	V1 = 2	0	
		V2 = 3	1	
		V3 = 4	0	
LVOT/LCC+RVOT/LPC	7	V1 = 2	0	Ablation at LPC was performed in two patients
		V2 = 4	0	
		V3 = 1	0	
LVOT+ GCV-AIV	2	V1 = 1	1	
		V2 = 1	0	
RVOT only	1	V3 = 1	0	
Percutaneous epicardial ablation after failure of endocardial ablation	2	V1 = 1	1	Endocardial ablation included LCC/LVOT
		V2 = 1	0	

LVOT—left ventricular outflow tract, LCC—left coronary cusp, RVOT—right ventricular outflow tract, LPC—left pulmonic cusp, GCV—great cardiac vein, AIV—anterior interventricular vein.

## Data Availability

Data are contained within this article.
